# Supplementary material for: Manipulating and monitoring nanoparticles in micellar thin film superstructures
Source: Nat Commun. 2018 Dec 6;9:5207. doi: 10.1038/s41467-018-07568-1 (PMC6283865; doi:10.1038/s41467-018-07568-1)
Supplement: Supplementary file 1 — Supplementary Information [file 41467_2018_7568_MOESM1_ESM.pdf]

Supplementary Information

Manipulating and Monitoring Nanoparticles in Micellar Thin Film Superstructures

Jan Bart ten Hove,[1,2] Fijs W.B. van Leeuwen,[1,2] and Aldrik H.Velders[*1,2]

1) Laboratory of BioNanoTechnology, Wageningen University & Research, Axis, Bornse Weilanden 9, 6708 WG Wageningen, The Netherlands

2) Interventional Molecular Imaging Laboratory, Department of Radiology, Leiden University Medical Centre, Leiden, The Netherlands

*) email: aldrik.velders@wur.nl

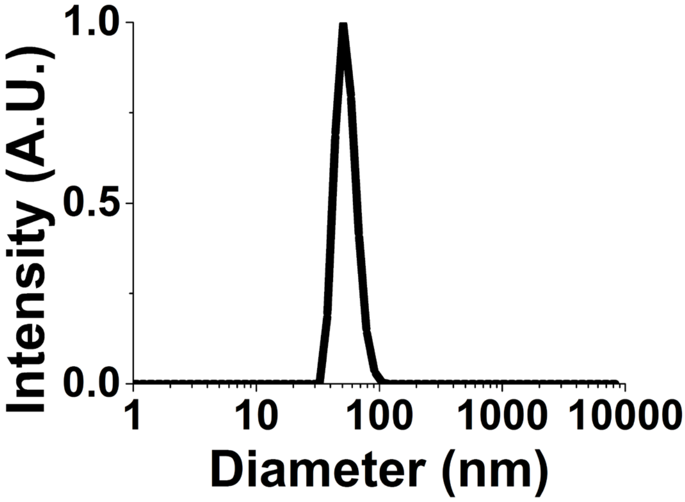

Supplementary Figure 1) DLS characterization of dendrimicelles. Number-averaged DLS size plot of dendrimicelles made (at charge-stoichiometry) from sixth generation poly(amidoamine) dendrimers hosting an Au_{128} nanoparticle within. The average hydrodynamic diameter was found to be 52 nm.

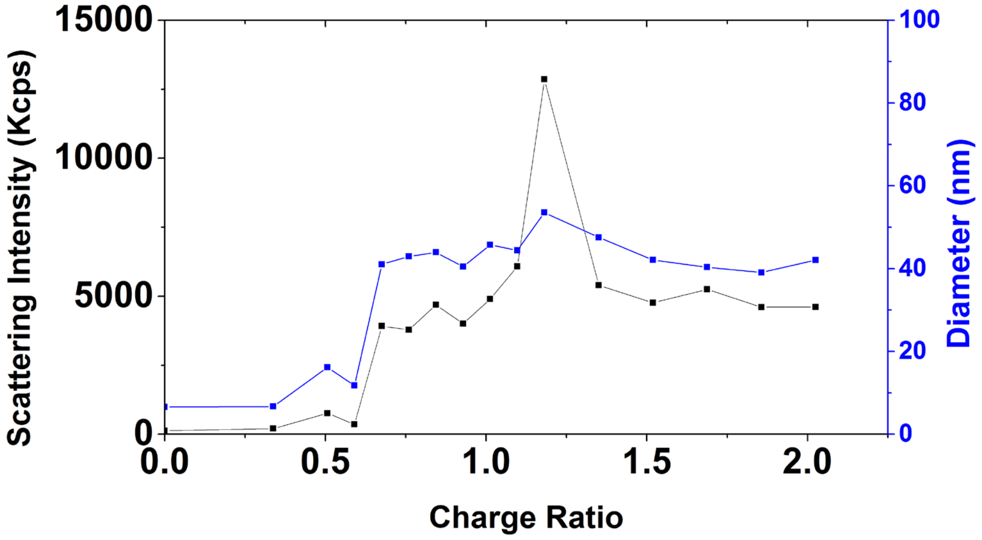

Supplementary Figure 2) DLS charge titration graph of PAMAM G6-NH_2 with $pMAA_{64}PEO_{885}$. The graph shows both the number-averaged dendrimicelle diameter (in blue) and the normalized scattered light intensity (in black) plotted against the charge fraction. The amount of positive charge (dendrimer-NH_2) was kept constant at 59 nmoles, and the amount of negative block polymer was varied, while keeping total volume constant. The charge fraction was calculated as the ratio of ($COOH/NH_2$).

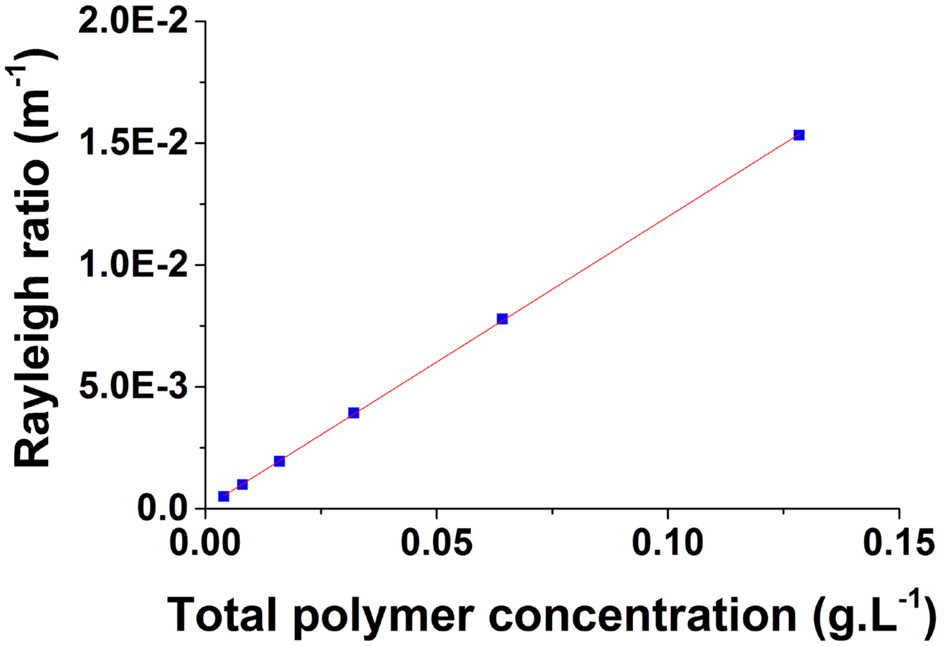

Supplementary Figure 3) CMC determination of dendrimicelles made from sixth generation poly(amidoamine) dendrimers. The intensity (as the excess Rayleigh ratio in m^{-1}) is plotted versus the total concentration of polymer (dendrimer + block copolymer). Fitting the data points with a linear fitting formula, and extrapolating to zero intensity gives the CMC. The thus-determined CMC is found at a negative polymer concentration (~-1 $mg.L^{-1}$), indicating the stability of the dendrimicelles. Since the linear fitting formula almost perfectly fits the data points (R^2>0.999), we attribute the negative sign of the CMC to experimental error, likely because the CMC lays below the limit of detection of our setup. Therefore, the CMC is at least lower than 4 $mg.L^{-1}$.

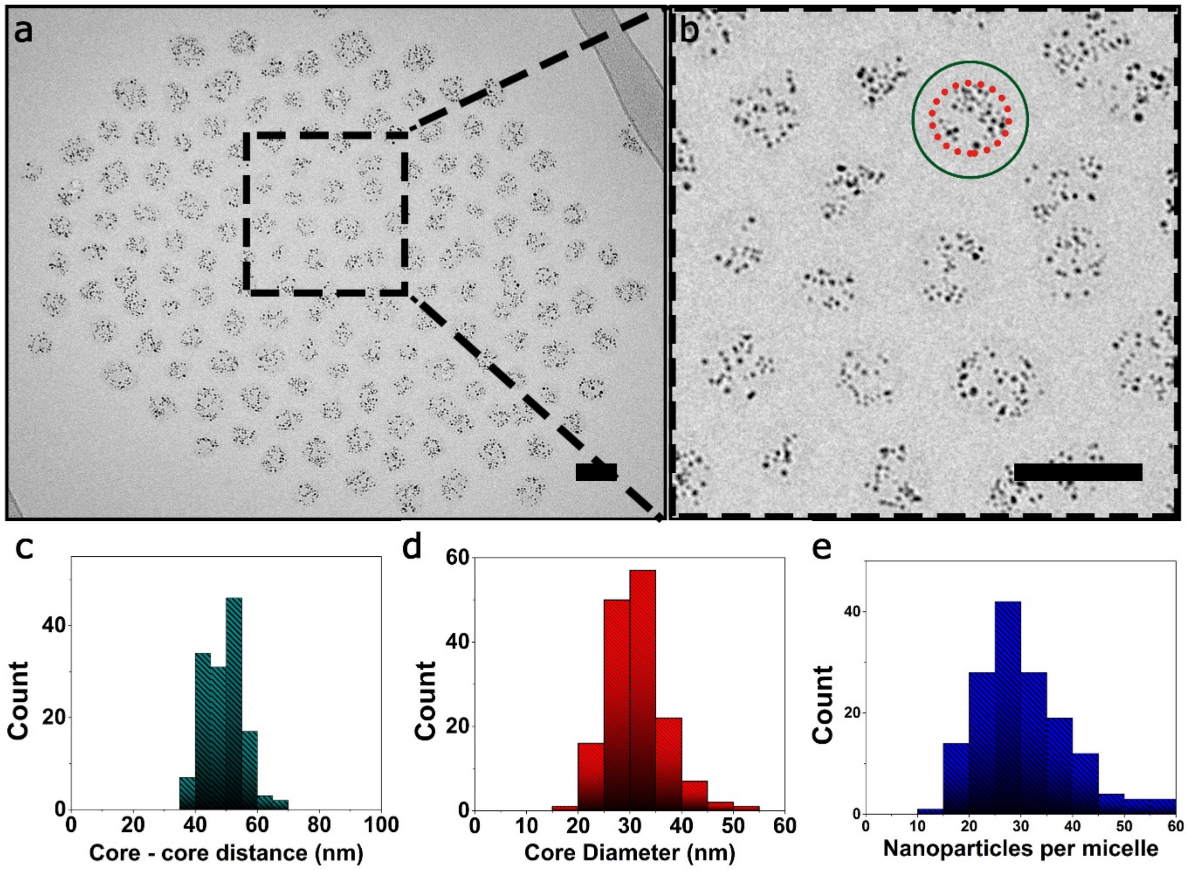

Supplementary Figure 4) Characterization of sixth generation-based dendrimicelles. **a)** cryoTEM micrograph of the formed dendrimicelle superstructure. **b)** Magnification of the superstructure. In this micrograph, the nanoparticles residing inside the dendrimicelle core (the black spots) reveal the location and size of the dendrimicelle core, as indicated with the red, dotted box. The green, solid, circle indicates the size of the total dendrimicelle (core+corona), as determined by DLS. Characterization of the cryoTEM images by measuring the core-core distances (**c**) shows that the average micelle is ~50 nm, has a core diameter that is ~30 nm (**d**), and contains about 30 ± 10 nanoparticles per dendrimicelle (**e**). Scale bars are 50 nm.

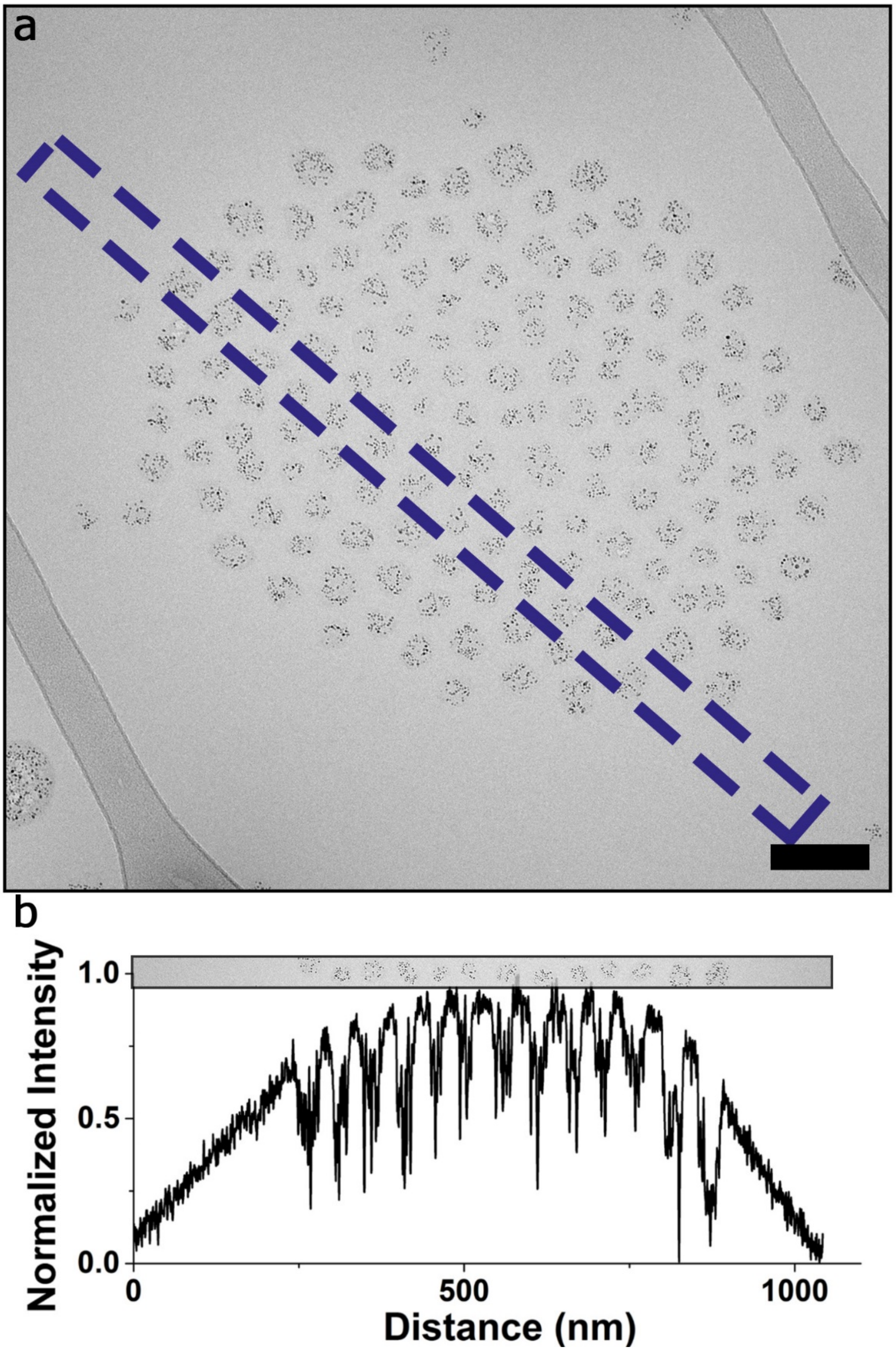

Supplementary Figure 5) PAMAM G6-based dendrimicelle superstructure analysis. **a)** TEM micrograph showing the superstructure. The blue, dotted, rectangle indicates the location in the micrograph where plot (b) has been calculated for. The scale bar represents 100 nm. **b)** By plotting the (normalized) image intensity versus distance, it is apparent that the amount of transmitted light is increased towards the center of the dendrimicelle superstructure, implying that the thickness of the water layer is thinner towards the center of the superstructure.

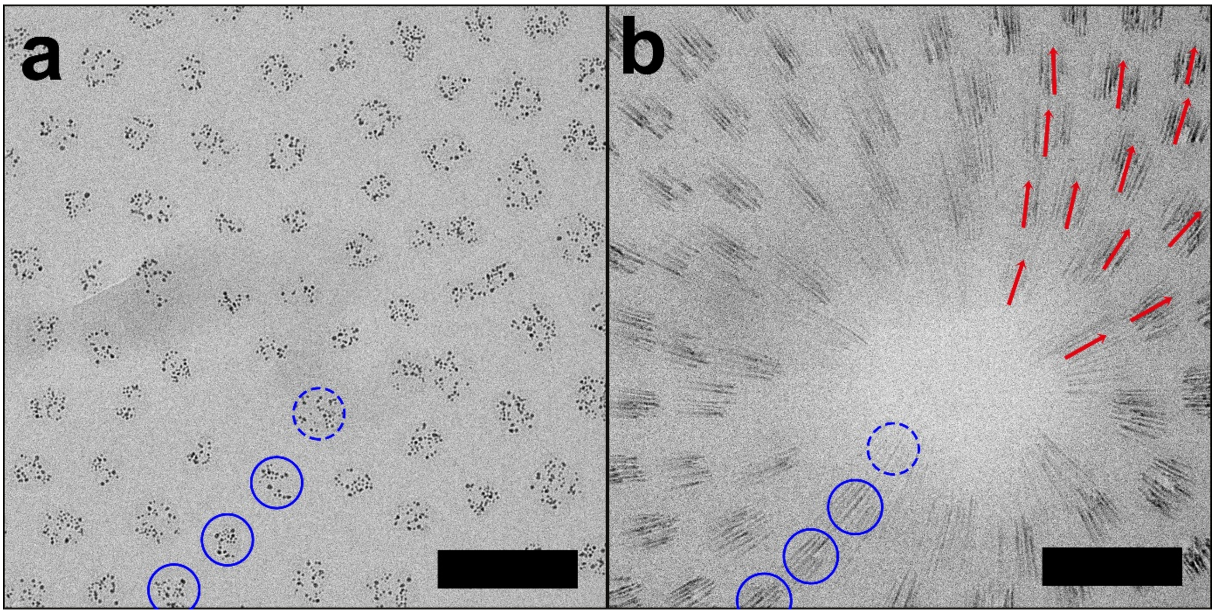

Supplementary Figure 6) Electron beam-induced thin film rupture of a sixth-generation PAMAM-based dendrimicelle superstructure. TEM micrographs showing: **a)** dendrimicelle superstructure and **b)** the rupture of the thin water film. Film rupture appears to start from the center of the superstructure, anisotropically moving outwards. Scale bars are 100 nm.

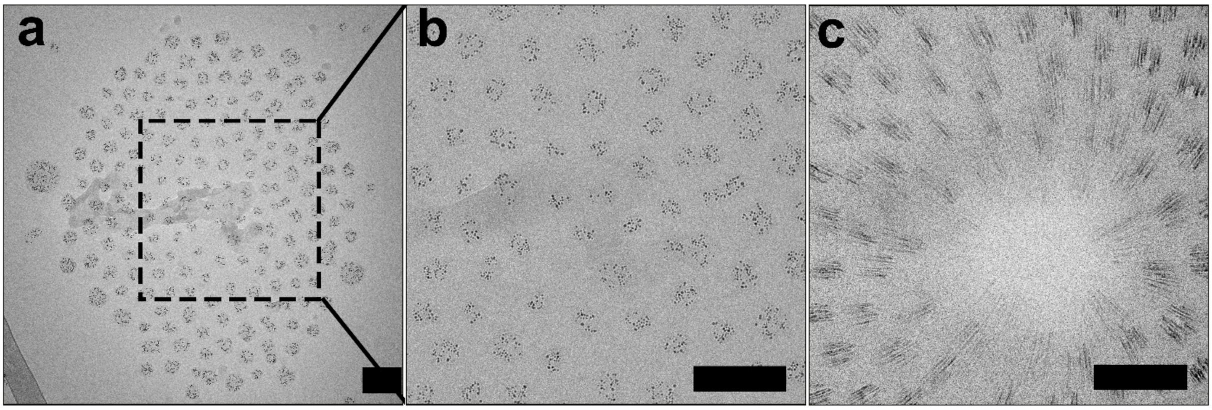

Supplementary Figure 7) Electron beam-induced thin film rupture of a sixth-generation PAMAM-based dendrimicelle superstructure. TEM micrographs showing: a) the dendrimicelle superstructure. b) Zoom-in on the center of the superstructure. c) electron-beam induced radiation damage, and d) the rupture of the thin water film. Although the structures toward the edge of the superstructure show the most signs of radiation damage, the film ruptures from the center of the superstructure. Scale bars are 100 nm.

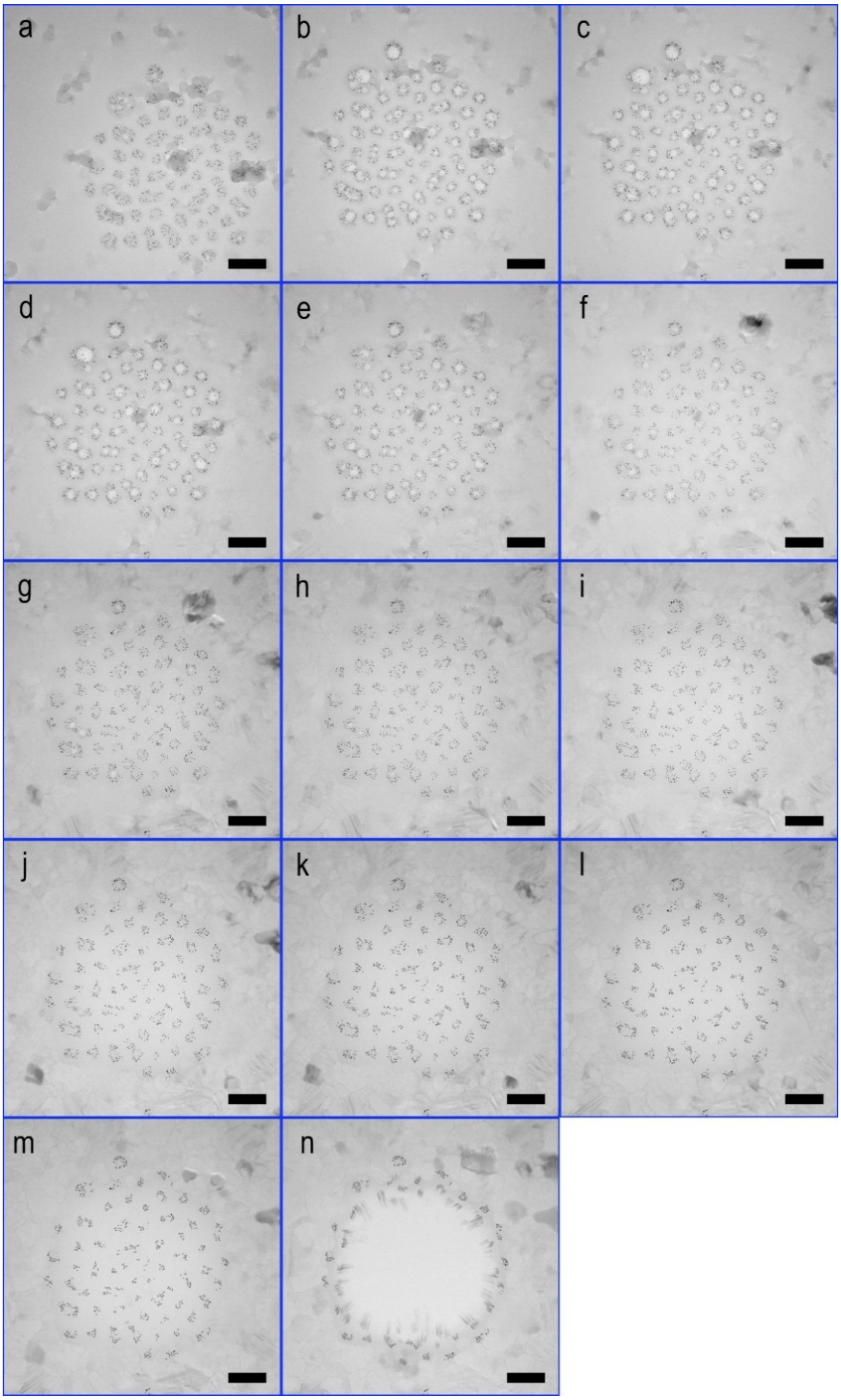

Supplementary Figure 8) Stroboscopic exposure of a generation six-based dendrimicelle superstructure. First, (b-d), radiation damage appears in the form of gas bubbles, followed by slow disappearance of the gas bubbles (e-h) and further evaporation of water (i-m). Finally, the superstructure ruptures, leaving a hole behind (n). The scale bars are 100 nm.

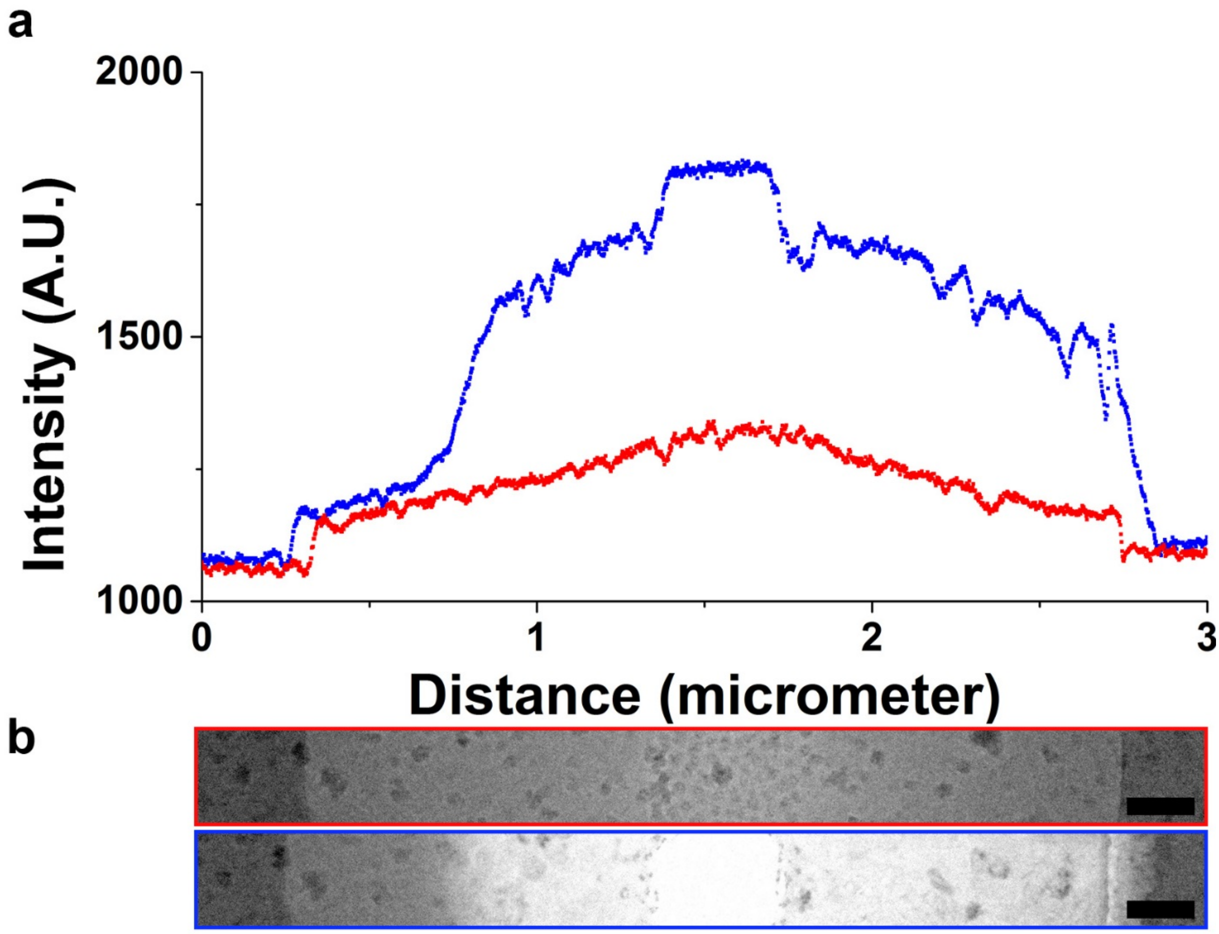

Supplementary Figure 9) Dendrimicelle superstructure characterization before and after stroboscopic exposure. Average image intensity profile plot (a) and corresponding cryoTEM micrographs (b) before (in red) and after (in blue) the stroboscopic exposure series. The location of the dendrimicelle superstructure coincided with the thinnest part of the biconcave thin film. The intensity plot depicts the image intensity averaged over the 0.3 µm tall micrograph, and shows that the stroboscopic exposure resulted in a decrease in film thickness. Scale bars are 200 nm.

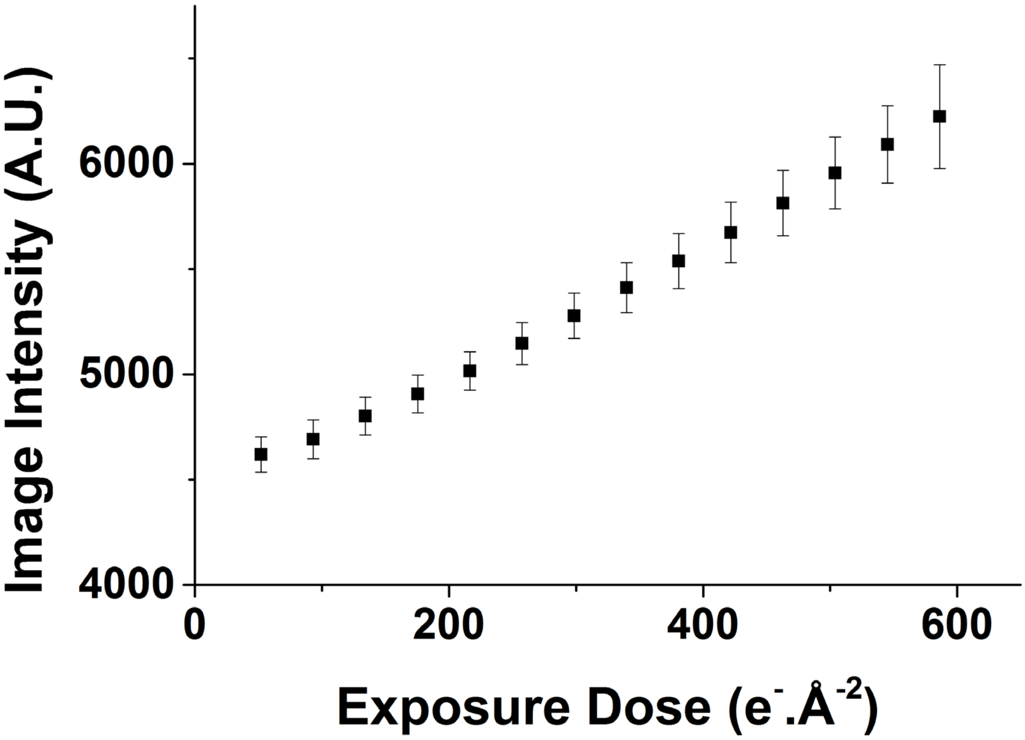

Supplementary Figure 10) Average image intensity versus electron exposure dose during stroboscopic exposure. The image intensity graph suggests a linear relationship between the exposure dose and transmission during the stroboscopic exposure series (as shown in Supplementary Figure 8). The error bars represent the standard deviation.

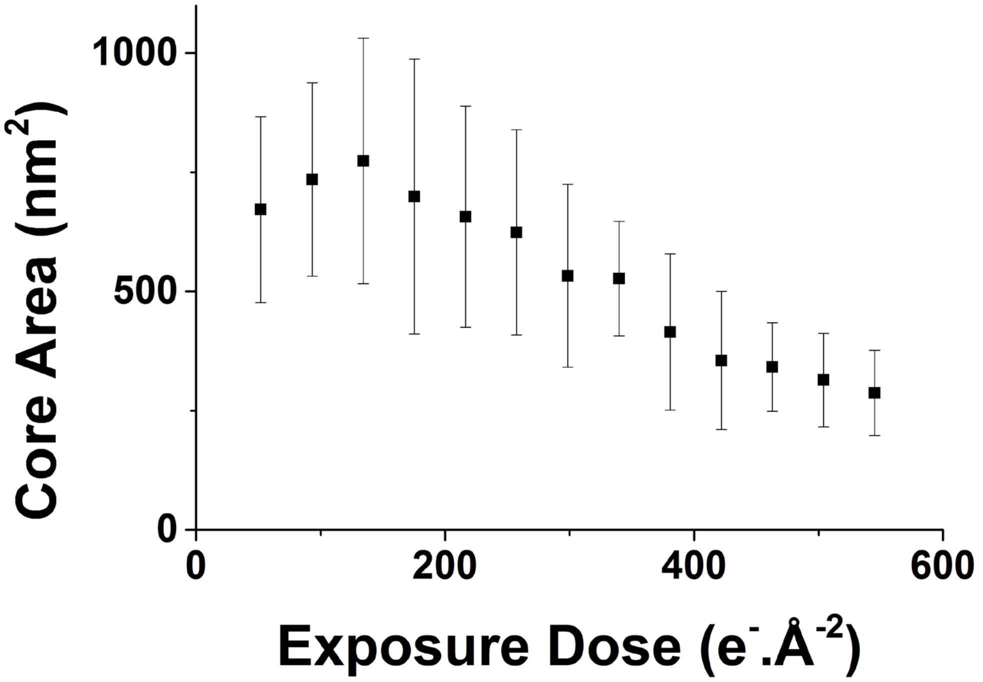

Supplementary Figure 11) Dendrimicelle core shrinkage during stroboscopic exposure. Measuring the average dendrimicelle core area—as outlined by the gold nanoparticles embedded within— of ten generation 6-based dendrimicelles (Supplementary Figure 8) indicates that the dendrimicelle core area decreased by ~40 percent during the during stroboscopic exposure series. This corresponds to a decrease in volume —assuming spherical dendrimicelles— by a factor of ~2. The error bars represent the standard deviation in dendrimicelle core area.

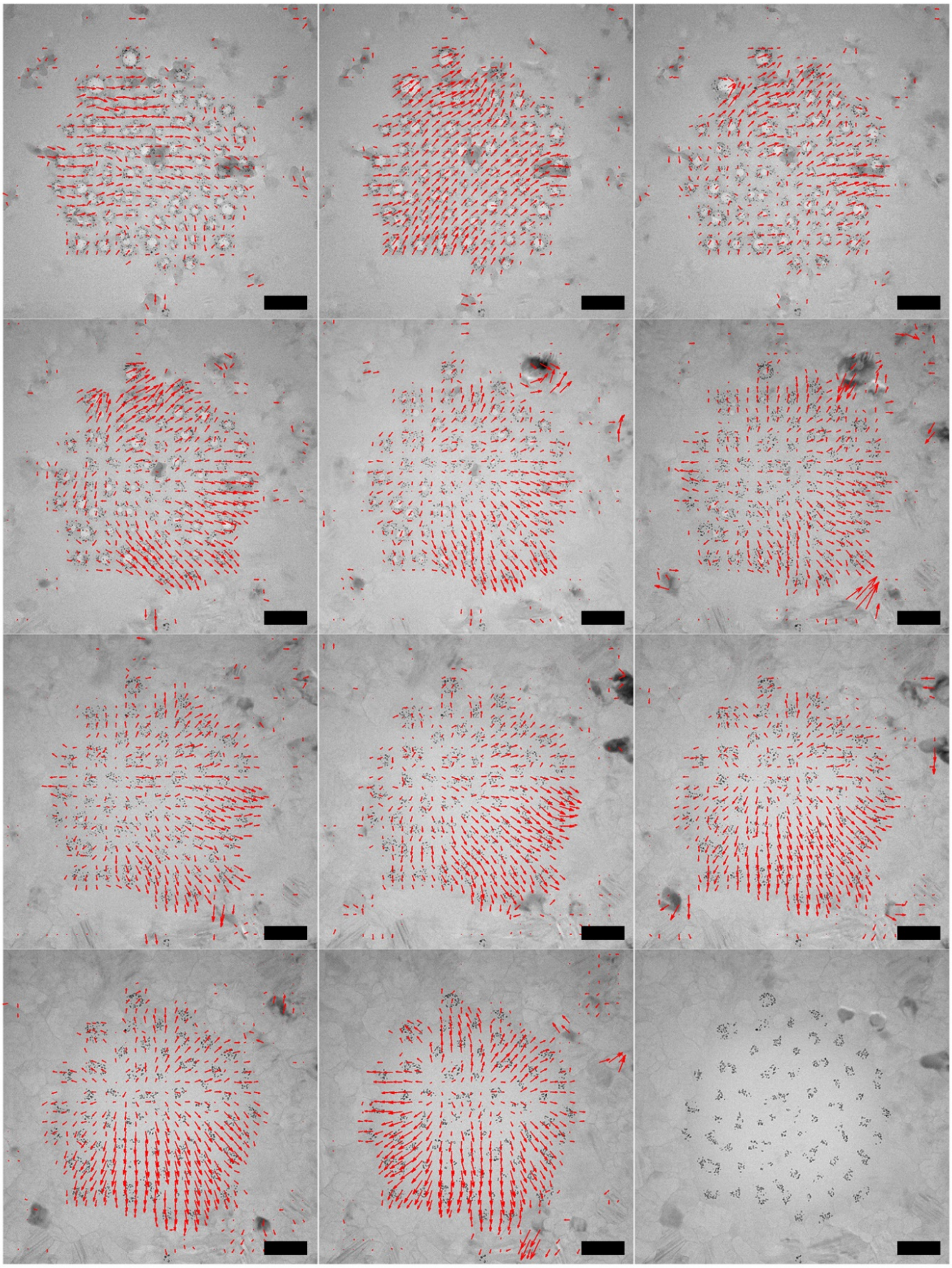

Supplementary Figure 12) Particle Image Velocimetry analysis results of the generation 6-based superstructure. The cryoTEM image sequence shown in Supplementary Figure 8 was used for the analysis. A window size of 64*64 pixels, corresponding to ~180*180 nm was used, with a spacing of 32 pixels. For clarity reasons, every other velocity vector is plotted. Scale bars are 100 nm.

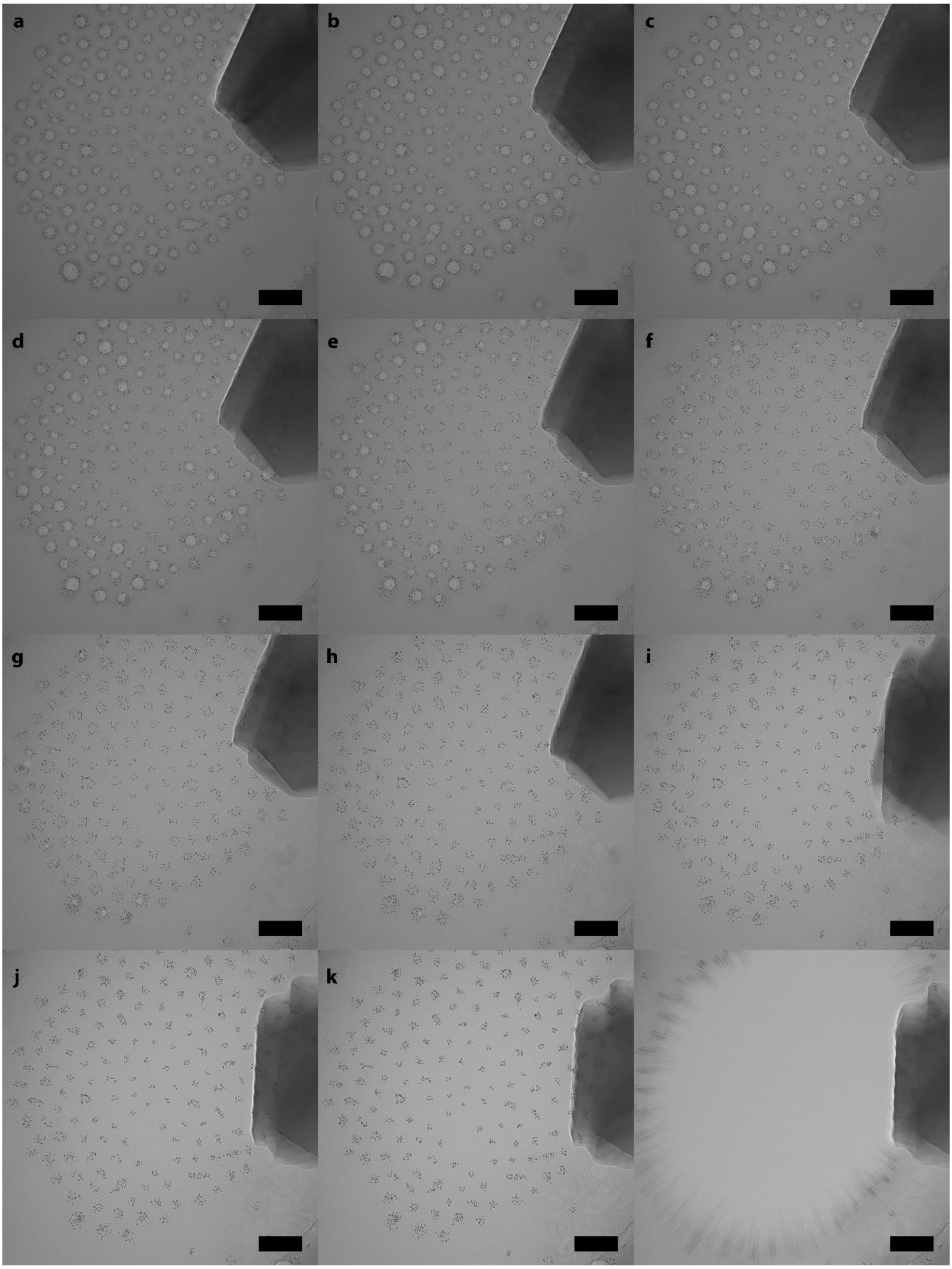

Supplementary Figure 13) Stroboscopic exposure series of a generation nine-based dendrimicelle superstructure. First (a-c), radiation damage appears in the form of gas bubbles, followed by the slow disappearance of the gas bubbles (d-g) and further evaporation of water (g-k). Finally, the superstructure ruptures, leaving a hole behind (l). Scale bars are 100 nm.

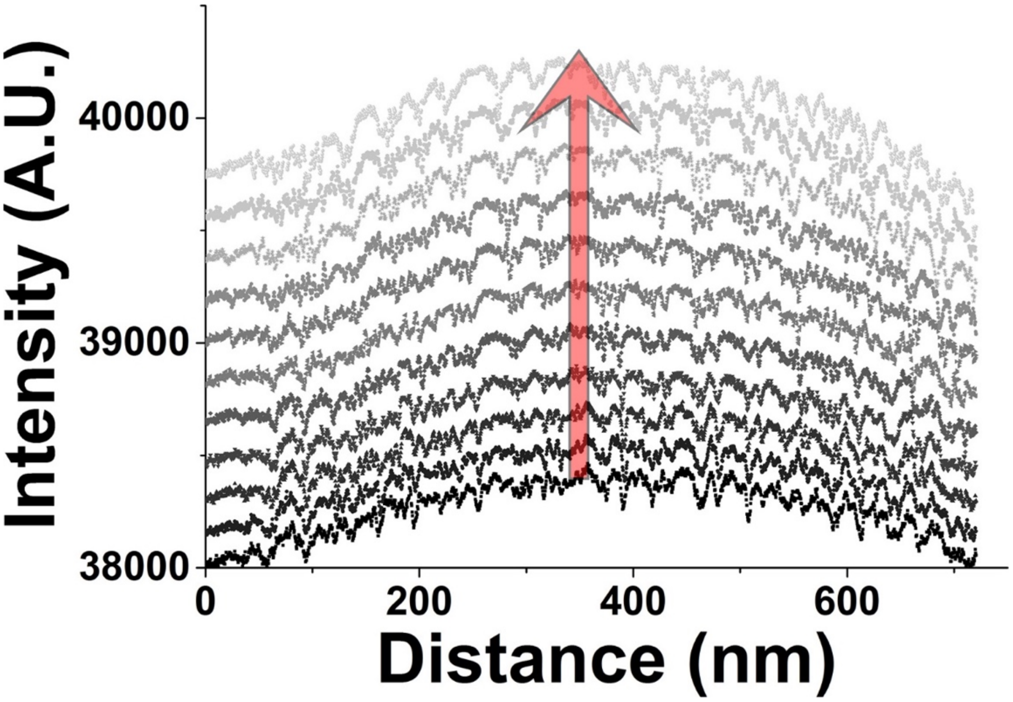

Supplementary Figure 14) Average intensity of the G9 dendrimicelle superstructure during the stroboscopic exposure. During the exposure series the average intensity increases, corresponding an increased transmission and hence a decrease in thin film thickness.

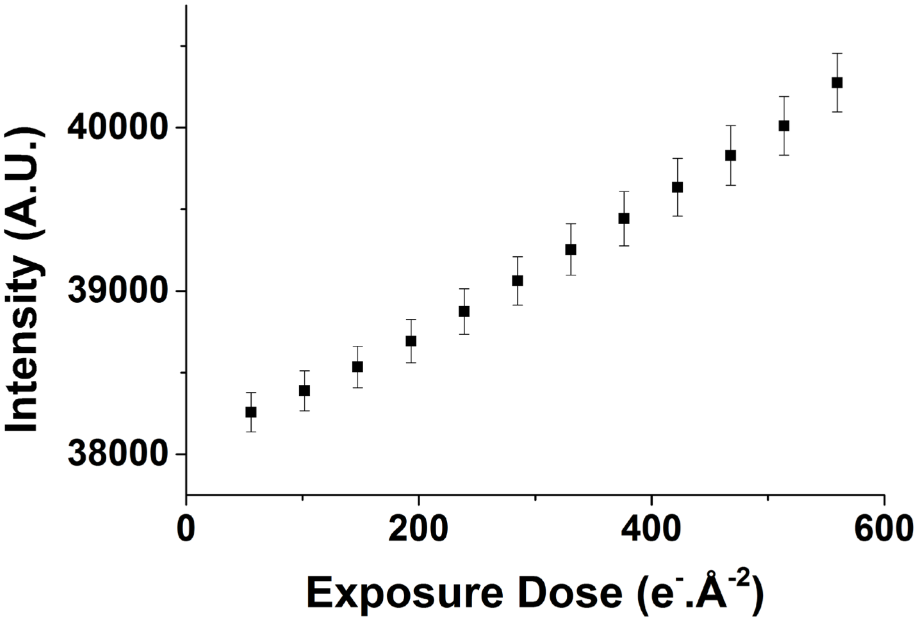

Supplementary Figure 15) Average image intensity versus electron exposure dose during stroboscopic exposure of the G9 dendrimicelle superstructure (as shown in Supplementary Figure 13). The graph shows an increase of the average intensity during the exposure series, implying an increased transmission and hence a decrease in thin film thickness. The error bars represent the standard deviation of the image intensity in an micrograph.

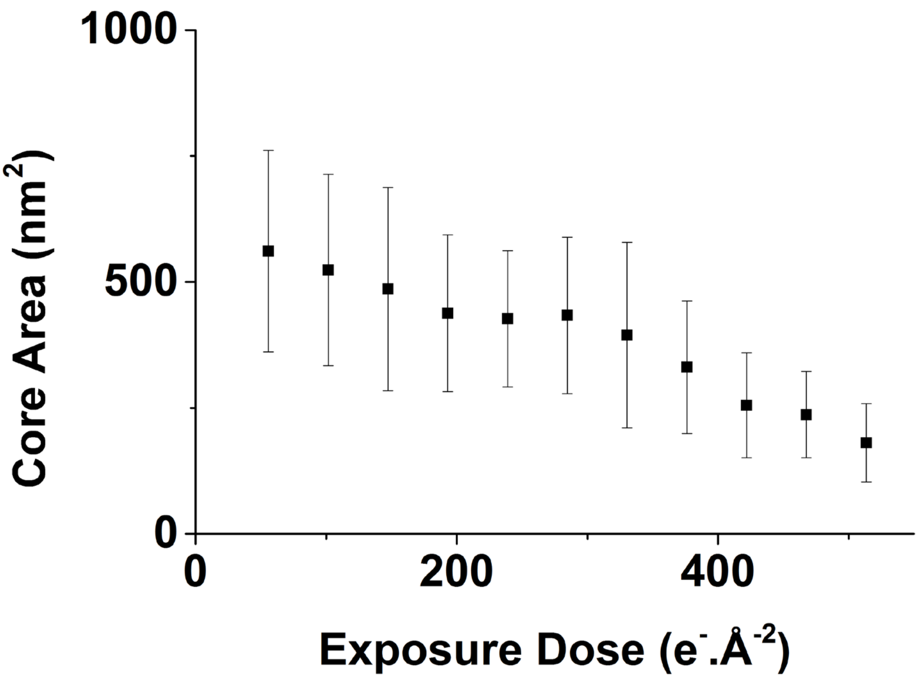

Supplementary Figure 16) Dendrimicelle core shrinkage during stroboscopic exposure. Analysis of the dendrimicelle core area —as outlined by the gold nanoparticles embedded within— of ten dendrimicelles in the G9 dendrimicelle superstructure (Supplementary Figure 13) indicates that the dendrimicelle core area decreased by ~70 percent during the during stroboscopic exposure series, suggesting the shrinkage of the dendrimicelle cores. This would correspond to a decrease in volume —assuming spherical dendrimicelles— by a factor of ~5. The error bars represent the standard deviation in dendrimicelle core area.

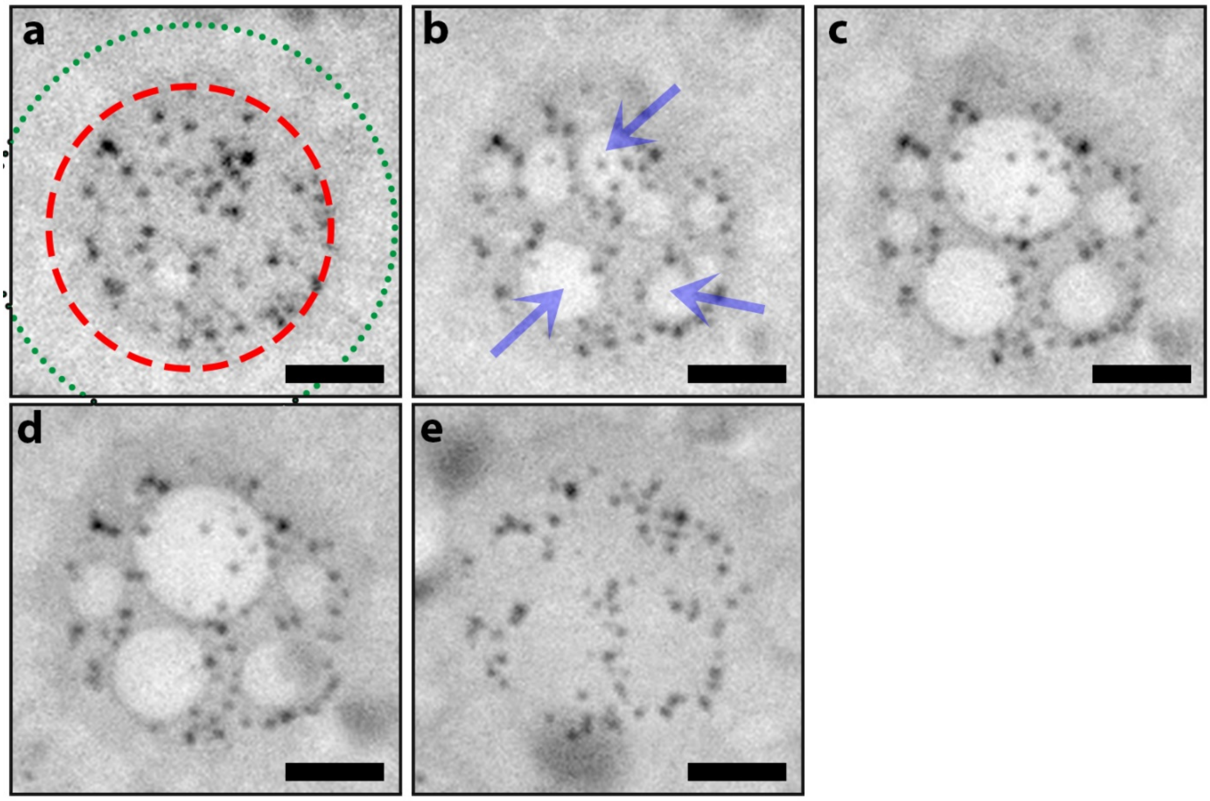

Supplementary Figure 17) Progressive electron beam-induced radiation damage to a PAMAM G6 dendrimicelle. The cryoTEM micrograph (**a**) shows the dendrimicelle core. Radiation damage appears as gas bubbles (**b**) (indicated with the blue arrows), that increase in size (**c-d**), and finally disappear (**e**). Upon further irradiation, the dendrimicelle superstructure ruptures, leaving a hole behind. The scale bars represent 10 nm.

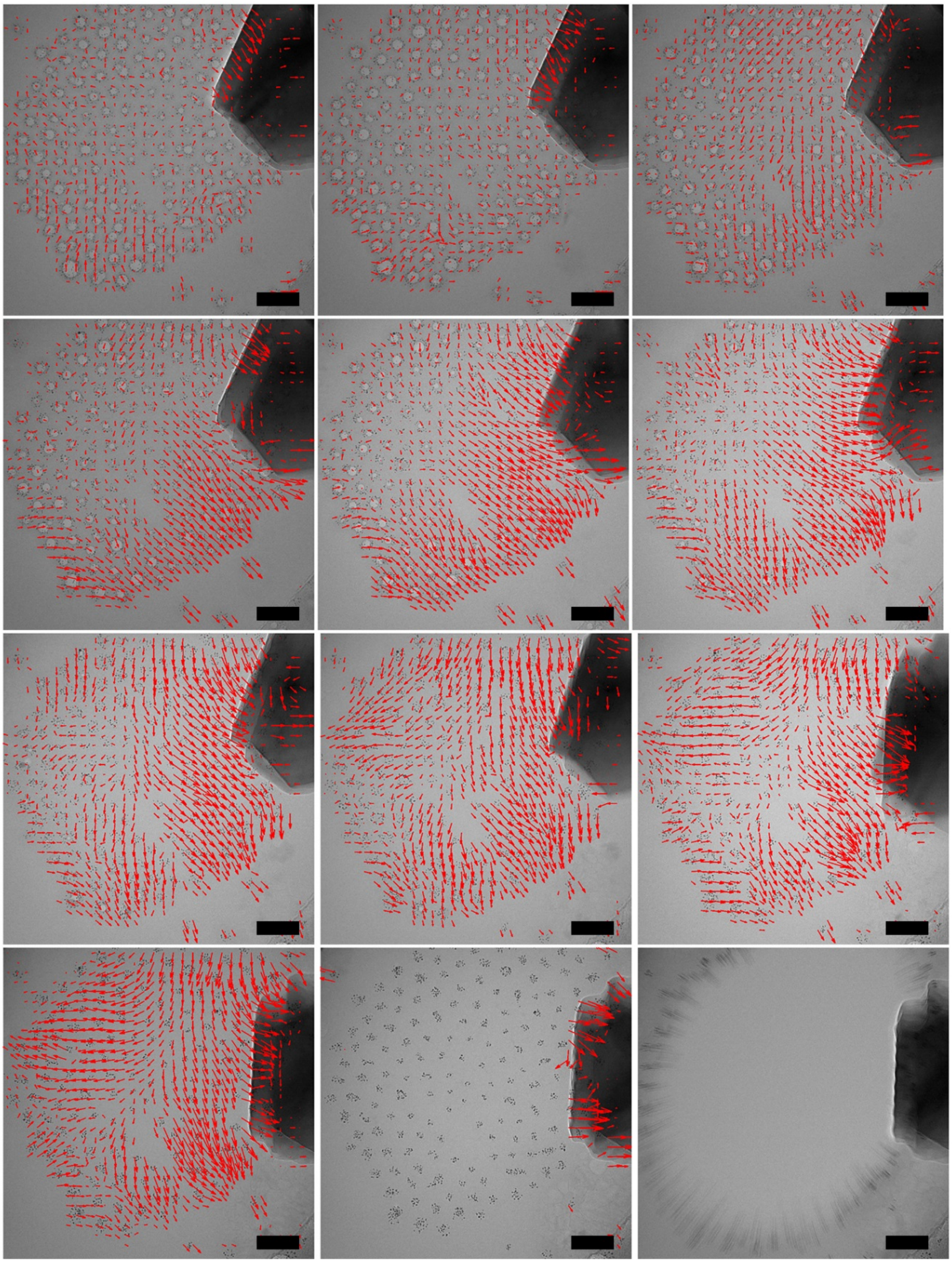

Supplementary Figure 18) Particle Image Velocimetry analysis results of the generation 9-based superstructure The image series as shown in Supplementary Figure 13 was used for this analysis. A window size of 64*64 pixels, corresponding to ~180*180 nm was used, with a spacing of 32 pixels. For clarity reasons, every other determined vector was plotted.

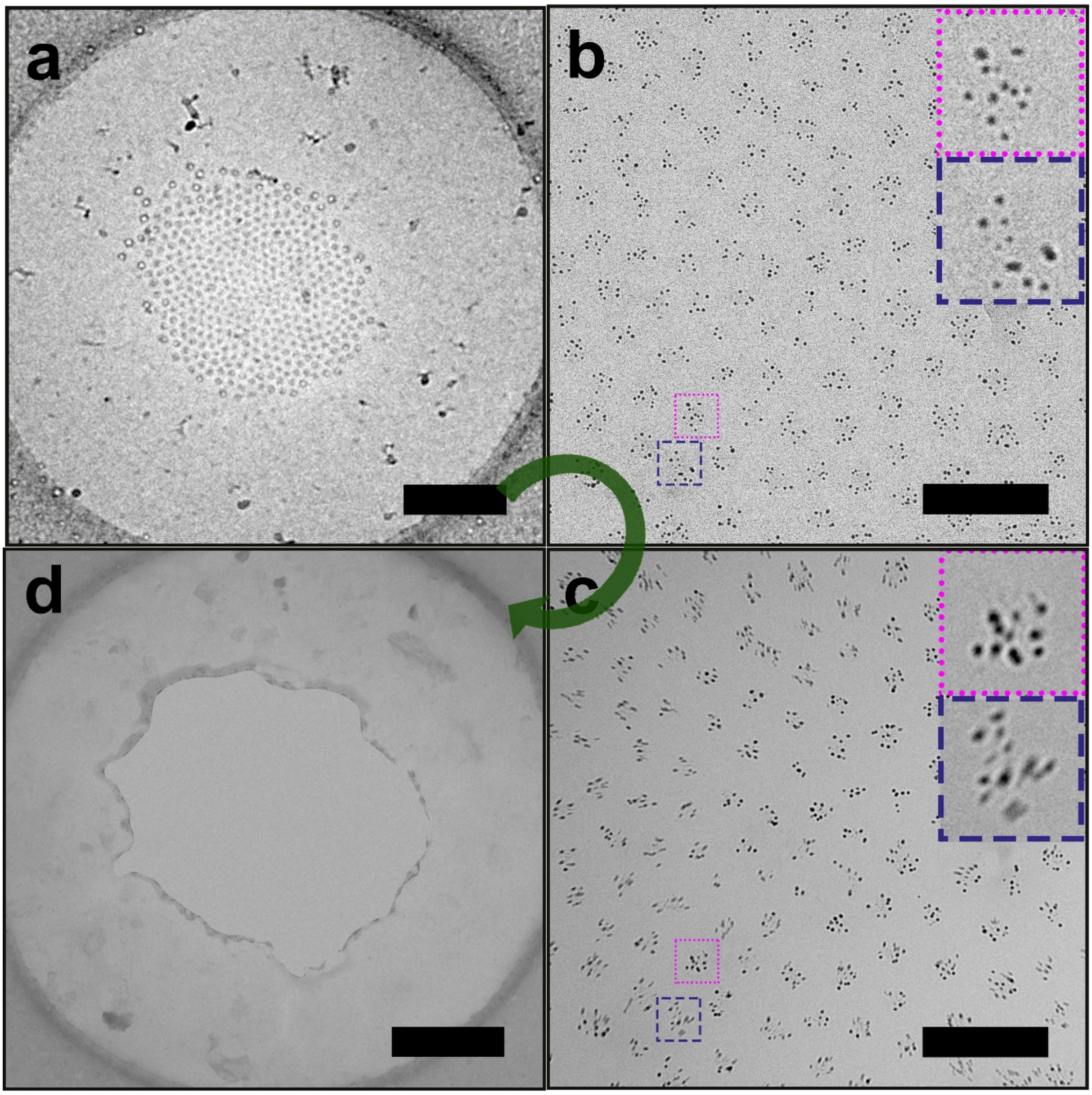

Supplementary Figure 19) Electron beam-induced rupture of biconcave thin water films containing a G9 dendrimicelle superstructure. a) Micron-sized hole in a circular grid hole, containing a vitrified, biconcave water film. b) Enlarged view of the dendrimicelle superstructure embedded in the biconcave thin film. c) the dendrimicelles just before rupture, with some of the dendrimicelles already showing local migration during acquisition in the form of motion blur. The pink, dotted inset box shows a dendrimicelle that remained static, and the purple, dashed, box shows a dendrimicelle that migrated during image acquisition, as indicated by the motion blur streaks. d) View of the TEM grid, showing the hole after electron beam-induced rupture. Scale bars are 500 nm (a/d), respectively 100 nm (b/c).

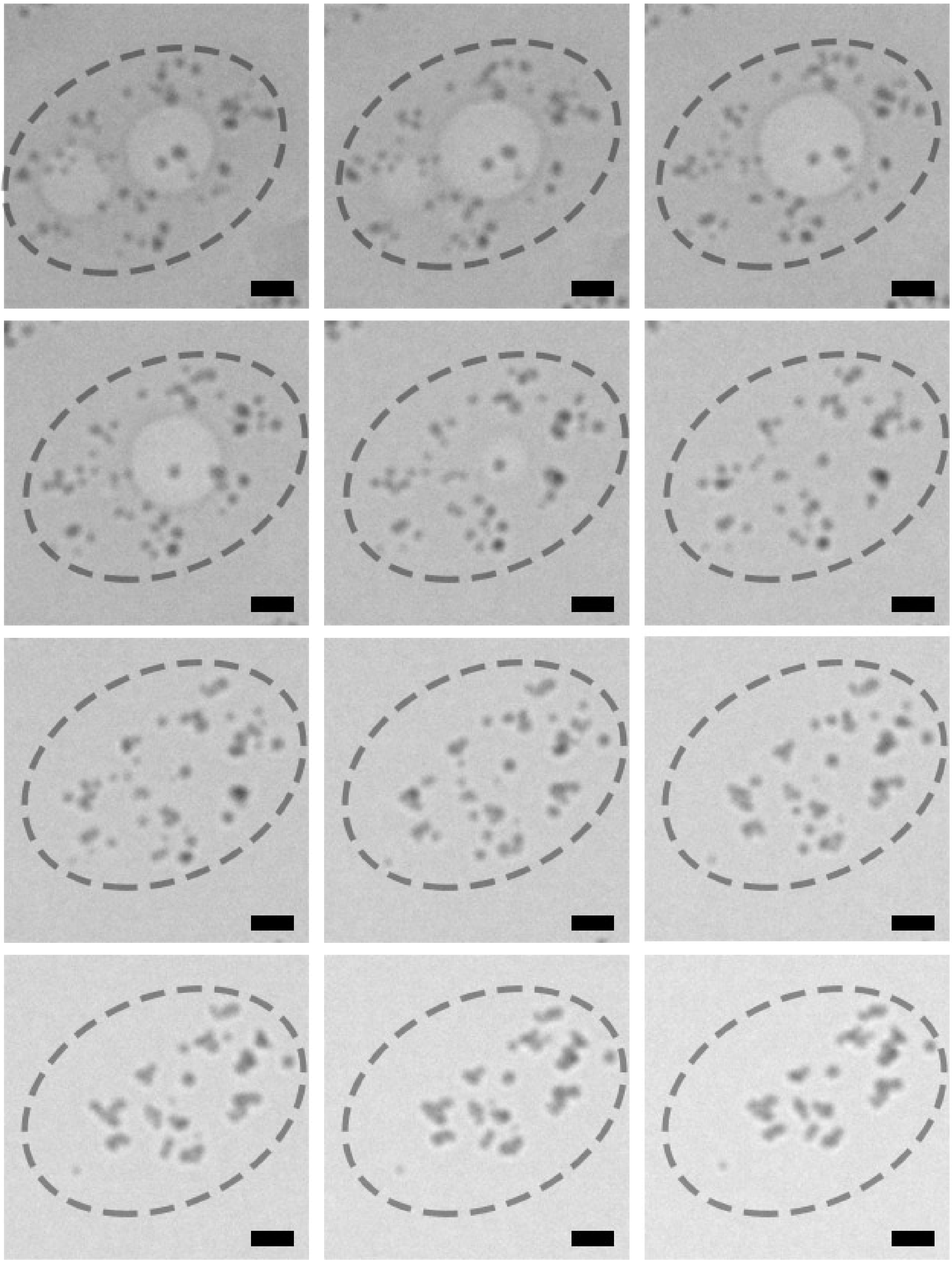

Supplementary Figure 20) Local motion inside dendrimicelle cores under stroboscopic exposure. CryoTEM micrographs (cropped from Supplementary Figure 8) of a single dendrimicelle under extended electron beam exposure, reveal not only the global migration of dendrimicelles, but also indicating the local migration of nanoparticles inside dendrimicelles. The dotted oval illustrates the dendrimicelle core shrinkage during the exposure series. Scale bars are 10 nm.
