# Supplementary material for: Manipulating and monitoring nanoparticles in micellar thin film superstructures
Source: Nat Commun. 2018 Dec 6;9:5207. doi: 10.1038/s41467-018-07568-1 (PMC6283865; doi:10.1038/s41467-018-07568-1)
Supplement: Supplementary file 3 — Description of Additional Supplementary Files [file 41467_2018_7568_MOESM3_ESM.pdf]

Description of Additional Supplementary Files

File Name: Supplementary Movie 1
Description: Stroboscopic exposure series of a generation six-based dendrimicelle superstructure. During the exposure series, first radiation damage is observed in the form of gas bubbles, followed by local (dendrimicelle core shrinkage) and global movement of the dendrimicelles inside the superstructure. Finally, the superstructure ruptures.

File Name: Supplementary Movie 2
Description: Stroboscopic exposure series of a generation nine-based dendrimicelle superstructure. During the exposure series, first radiation damage is observed in the form of gas bubbles, followed by local (dendrimicelle core shrinkage) and global movement of the dendrimicelles inside the superstructure. Finally, the superstructure ruptures.
